# Quantifying Charge Carrier Recombination Losses in MAPbI_3_/C60 and MAPbI_3_/Spiro-OMeTAD with and without Bias Illumination

**DOI:** 10.1021/acs.jpclett.2c01728

**Published:** 2022-08-10

**Authors:** V.M. Caselli, T.J. Savenije

**Affiliations:** Department of Chemical Engineering, Delft University of Technology, van der Maasweg 9, 2629 HZ Delft, The Netherlands

## Abstract

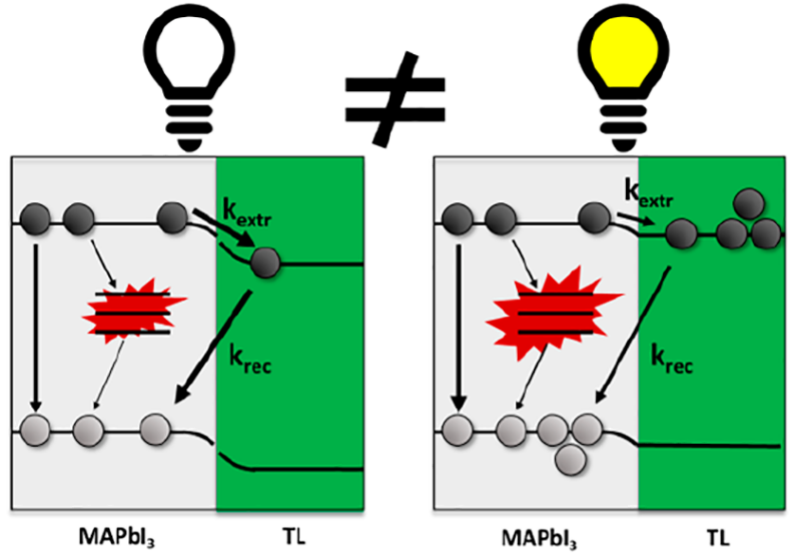

To increase the open-circuit voltage in perovskite-based solar cells, recombination processes at the interface with transport layers (TLs) should be identified and reduced. We investigated the charge carrier dynamics in bilayers of methylammonium lead iodide (MAPbI_3_) with C60 or Spiro-OMeTAD using time-resolved microwave conductance (TRMC) measurements with and without bias illumination (BI). By modeling the results, we quantified recombination losses in bare MAPbI_3_ and extraction into the TLs. Only under BI did we find that the density of deep traps increases in bare MAPbI_3_, substantially enhancing trap-mediated losses. This reversible process is prevented in a bilayer with C60 but not with Spiro-OMeTAD. While under BI extraction rates reduce significantly in both bilayers, only in MAPbI_3_/Spiro-OMeTAD does interfacial recombination also increases, substantially reducing the quasi Fermi level splitting. This work demonstrates the impact of BI on charge dynamics and shows that adjusting the Fermi level of TLs is imperative to reduce interfacial recombination losses.

Metal halide perovskite-based solar cells (PSCs) have improved significantly over the past years, reaching device efficiencies over 25%.^1^ This impressive progress can be attributed to different optimization procedures. The perovskite layer properties have been perfected by optimizing the synthesis and deposition methods to obtain more stable and highly crystalline perovskite layers.^1−4^ At the device level, many approaches have been proposed to improve the interface properties between the perovskite absorber layer and the selective transport layers (TLs).^5^ Poor band alignment, defect states at the interfaces, and instability of the used transport materials lead to a reduction in device performance, typically in the form of a reduction of the open-circuit voltage (*V*_oc_).^6^ To overcome these issues, many different materials, organic and inorganic, have been examined as electron or hole transport layers.^7−11^ However, for rational design of efficient perovskite-based solar cells with a high *V*_oc_, it is essential to obtain information regarding the rates for the charge extraction and recombination processes occurring at the perovskite/TL interfaces.

Different experimental methods have been used to characterize the interface of the perovskite layer with the TLs.^5,8,12,13^ Increasing photoluminescence yields and enhanced device stabilities and efficiencies have been often related to improved interfacial properties. Nonetheless, only a few research groups have been able to provide a quantitative analysis of the rate constants for charge extraction and interfacial recombination.^14−19^ In all these studies, the charge carrier dynamics have been investigated by means of time-resolved techniques using pulsed illumination sources. However, the kinetic parameters that can be extracted from a time-resolved analysis are not always representative of the dynamics under steady-state illumination.^20^ In addition, it has been reported that, for example, MAPbI_3_ is unstable under continuous illumination. More specifically, ion migration has been found to be the cause of several instability issues and hysteresis in the *J*–*V* curves of MAPbI_3_-based devices.^5,7,9,21^ It can be expected that ion migration influences not only the charge carrier dynamics in the perovskite layer but also the efficiency of the charge extraction process by the TLs.^7,22^ For these reasons, quantitatively studying the charge carrier extraction in perovskite/TL bilayers under steady-state illumination is relevant to fully characterize the interfacial processes.

In this Letter we provide a quantitative analysis to extract the rates of charge carrier extraction and recombination processes for methylammonium lead iodide (MAPbI_3_) with selective TLs (see Scheme 1) under bias illumination (BI). C60 and Spiro-OMeTAD are chosen as electron transport layer (ETL) and hole transport layer (HTL), respectively, as they are the most commonly applied materials in the corresponding p-i-n and n-i-p cell structures. The dynamics are revealed by time-resolved microwave photoconductance (TRMC) measurements in the presence or absence of continuous bias illumination with an intensity comparable to 0.3 suns. The analysis has been carried out by first investigating the optoelectronic properties of the bare MAPbI_3_ layer, followed by the MAPbI_3_/C60 and MAPbI_3_/Spiro-OMeTAD bilayers. The contactless TRMC measurements are performed over a broad intensity range from 10^13^–10^15^ cm^–3^, allowing us to quantify the various rate constants (see Scheme 1a). Unless specified otherwise, the samples have been illuminated from the quartz side, referred to as the back side (BS) in Scheme 1b, in order to reduce parasitic absorption of BI by the TLs.

**Scheme 1:**
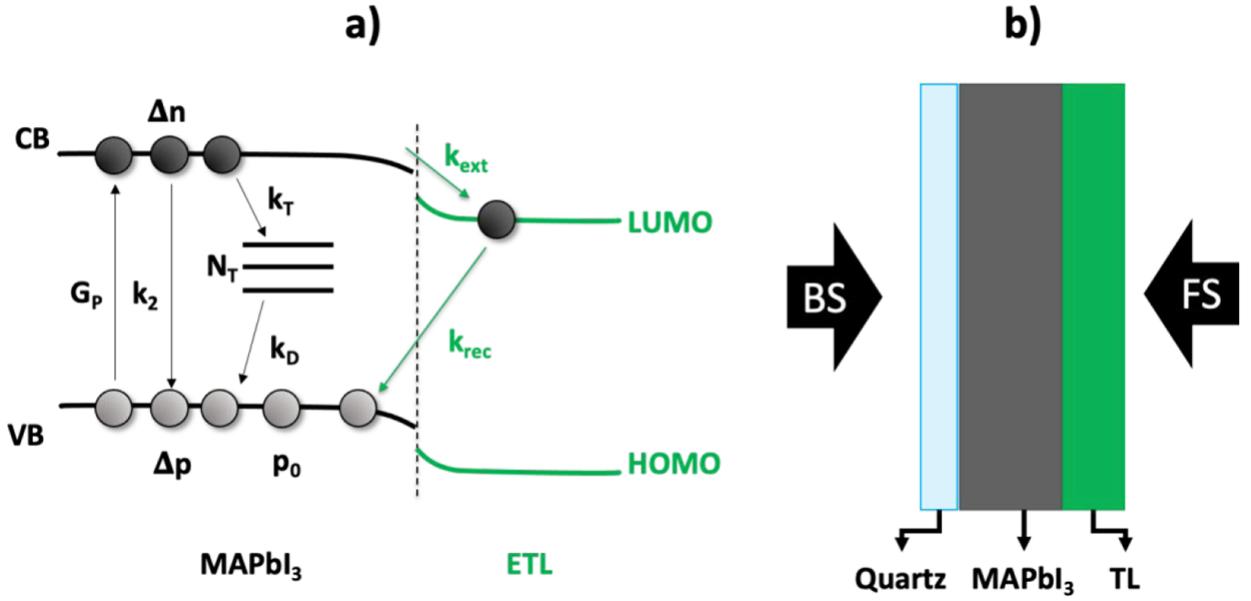
(a) Schematic Representation of a MAPbI_3_/ETL Heterojunction and Relevant Kinetic Processes Occurring under Illumination; (b) Schematic Representation of the Sample Configuration and Excitation Side In panel a, the processes in the MAPbI_3_ film are shown in black, while the charge carrier extraction and back recombination due to the presence of the ETL are shown in green. In panel b, front side (FS) is performed by illuminating the sample from the perovskite/TL side, back side (BS) excitation by illuminating though the quartz substrate

Confirming previous findings, we observe in MAPbI_3_/C60 bilayers fast and efficient charge extraction by the C60 layer on pulsed illumination.^8−10,23^ However, while a bare MAPbI_3_ layer shows fully reversible degradation under BI, the introduction of C60 suppresses this degradation process. On the other hand, the MAPbI_3_/Spiro-OMeTAD bilayer is characterized by the formation of an internal electric field affecting the charge extraction. Furthermore, under BI the concentration defect states at the MAPbI_3_/Spiro-OMeTAD interface increases substantially, which leads to partially irreversible changes in charge carrier dynamics. Finally, we calculate the concentrations of carriers under BI using the found kinetic parameters extracted from the TRMC analysis.^24^ From these concentrations, quantification of the recombination losses and the quasi Fermi level splitting corresponding to the upper limit of *qV*_OC_ of a device are determined and discussed.

MAPbI_3_ thin films have been spin-coated onto quartz substrates following a previously reported procedure.^23^ Optical and morphological characterizations are reported in the Supporting Information. A 30 nm thick C60 layer has been added via physical vapor deposition, while Spiro-OMeTAD has been spin-coated on top of the MAPbI_3_ film, as described in the Supporting Information. First, the TRMC signals observed upon 650 nm photoexcitation of single and bilayers of MAPbI_3_ (red) and MAPbI_3_/TL (with ETL in green and HTL in orange) without BI are shown in Figure 1a,b. Two characteristics can be observed: the magnitude of the TRMC signal of the bilayers is reduced with respect to the signal of the bare MAPbI_3_, and at the same time, part of the photoconductance signal shows a slower decay.^23^ The present results are in qualitative agreement with the data on single and double layers reported previously.^23^ Both these effects can be explained by efficient charge carrier extraction by the TLs. Basically, the TRMC signal is proportional to the concentration of charge carriers, *n*_*i*_, times their mobility, μ_*i*_, as given in eq 1:
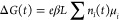
1Here, *e* is the elementary charge, β a dimensionality factor, and *L* the sample thickness. As the electron and hole mobilities in the TLs are more than 1 order of magnitude lower than in the perovskite layer, the extracted carriers have a negligible contribution to the measured photoconductance signal.^25,26^ This results in lower signals for the bilayers compared to the bare MAPbI_3_ layer. Furthermore, if efficient extraction occurs, the charge concentration of one type of carrier in the bands is less, reducing the decay by (non) radiative second-order recombination, and thus, long-lived photoconductance signals are observed.

**Figure 1:**
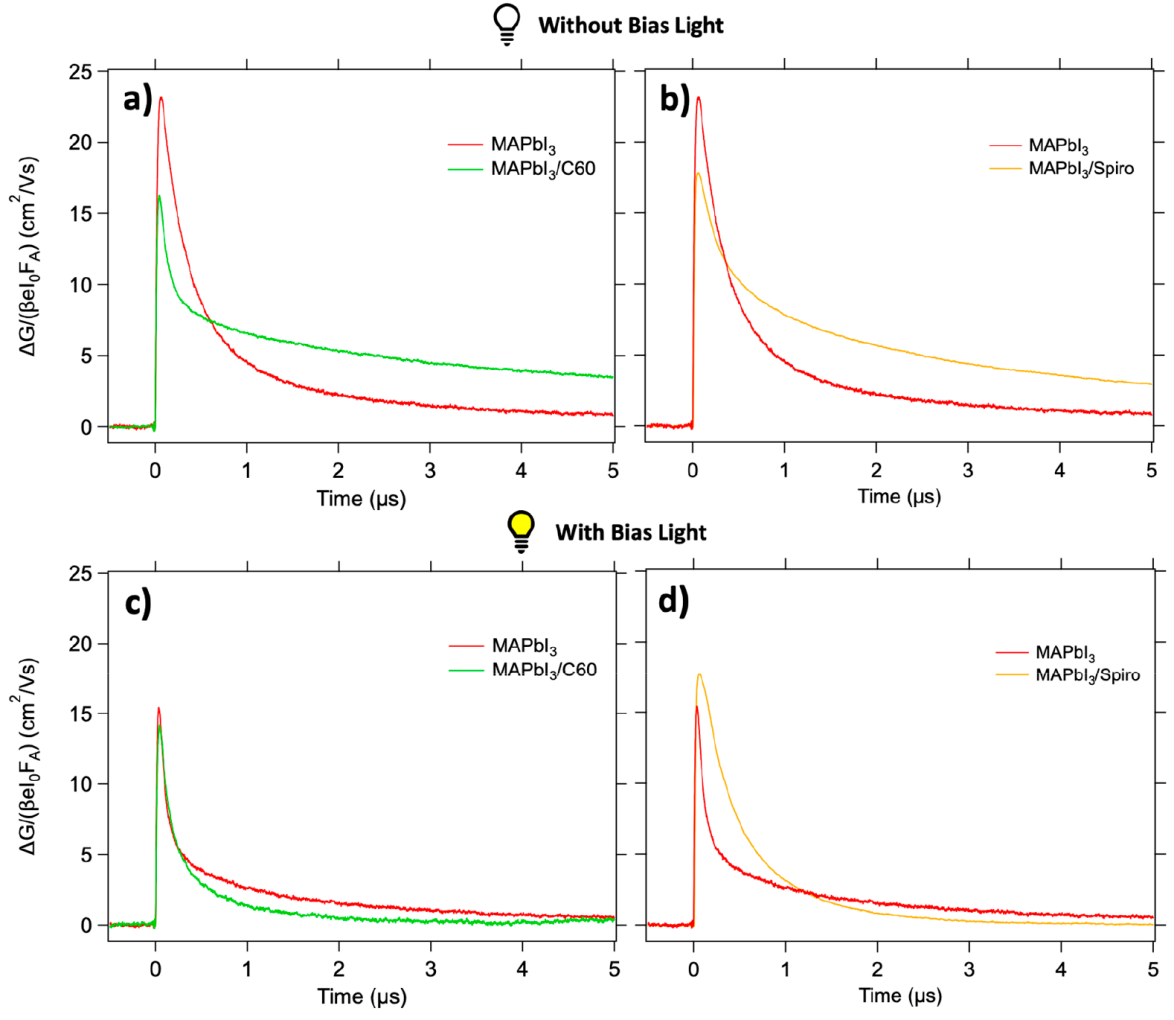
TRMC results upon excitation at 650 nm yielding an initial excitation density of 10^15^ charges/cm^3^/pulse for MAPbI_3_ (red), MAPbI_3_/C60 (green), and MAPbI_3_/Spiro-OMeTAD (orange) in absence (a and b) and presence (c and d) of a 0.3 sun bias illumination.

The TRMC results obtained under BI are shown in Figure 1c,d, displaying overall faster decays. This is expected because the higher excess charge carrier concentrations under BI enhance second-order recombination. Most interestingly, the dynamics in the MAPbI_3_/C60 bilayer is almost identical to that in the MAPbI_3_ single layer, as is evident from Figure 1c, suggesting reduced charge extraction. Also, for the MAPbI_3_/Spiro-OMeTAD bilayer, faster decays are observed on BI and the decay does not show any long-lived tail. Nonetheless, in comparison to the single MAPbI_3_ layer under BI, the lifetimes for the bilayer are still substantially longer (see Figure 1d). This might imply that part of the excess carriers is still extracted by the Spiro-OMeTAD, although less extensively than without BI. As shown in Figure S5, similar trends can be observed upon FS excitation of the bilayers. The independence of the excitation side implies that neither the initial excitation profile nor the period involved with diffusion of charges through the MAPbI_3_ dominates the decay kinetics on the used time scales.

In order to deduce the charge carrier extraction rates with and without BI, we adapted the kinetic model presented by Hutter et al.^27^ The model accounts for all the processes provided in Scheme 1a, and it allows us to determine the time-dependent charge carrier concentrations by solving four coupled differential eqs (eqs 2–5 for perovskite/ETL heterojunctions, and equations S2–S5 in the Supporting Information for a perovskite/HTL bilayer).
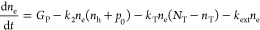
2
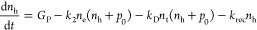
3
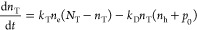
4
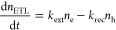
5

These equations include generation and recombination terms for electrons, *n*_e_, and holes, *n*_h_. *G*_P_ is the pulsed generation term; *k*_2_ is the second-order rate constant; and *k*_T_ and *k*_D_ are the trapping and detrapping rate constants, respectively. It is important to note that the second-order recombination revealed by TRMC measurements accounts for both radiative and nonradiative contributions, as discussed in a previous study.^28^ These parameters, together with the total number of trap states, *N*_T_, and background carrier concentration, *p*_0_, are characteristic of spin-coated MAPbI_3_. Because of the p-type character of MAPbI_3_, *n*_0_ is negligible, and this term is left out in the coupled differential equations. Charge carrier extraction is characterized by the extraction and back recombination rate constants, *k*_ext_ and *k*_rec_, respectively.

Typically, TRMC results are modeled taking into account the temporal laser pulse profile and its intensity (Figure 2a), resulting in the *G*_P_ term used in eqs 2 and 3. The modeled photoconductance signals, fits to the TRMC traces, result from the sum of the time-dependent electron and hole contributions times their individual mobilities at specific intensities. In Figure 2b, the modeled electron (orange), hole (red), and trapped electron (green) concentrations are shown for the specific case of 9.8 × 10^13^ excitations/cm^3^/pulse of the bare MAPbI_3_ layer. In the next step we use the kinetic parameters obtained from the TRMC analysis to model the concentration of electrons and holes in the perovskite material under BI.^24^ This is achieved by replacing the temporal profile of the laser pulse with a continuous illumination profile, *G*_Bias_ as shown in Figure 2c (see the Supporting Information for calculation of *G*_Bias_). The results for the MAPbI_3_ single layer, presented in Figure 2d, show that an equilibrium is reached in the perovskite film within 6 μs, after which electron and hole concentrations remain constant. In order to model the laser pulse-induced TRMC traces under BI, both the continuous bias illumination and laser pulse are combined in the generation term, *G*_Com_, as shown in Figure 2e. The calculated concentration profiles from the model are shown in the inset of Figure 2f. Since the TRMC setup records only the AC part of the photoconductance, the modeled traces are vertically shifted, providing only the AC contributions as exemplified in Figure 2f (see Experimental Methods for details) These calculated traces show the characteristic decays of a pulsed laser experiment but take into account the charge carrier concentrations induced by the BI.

**Figure 2:**
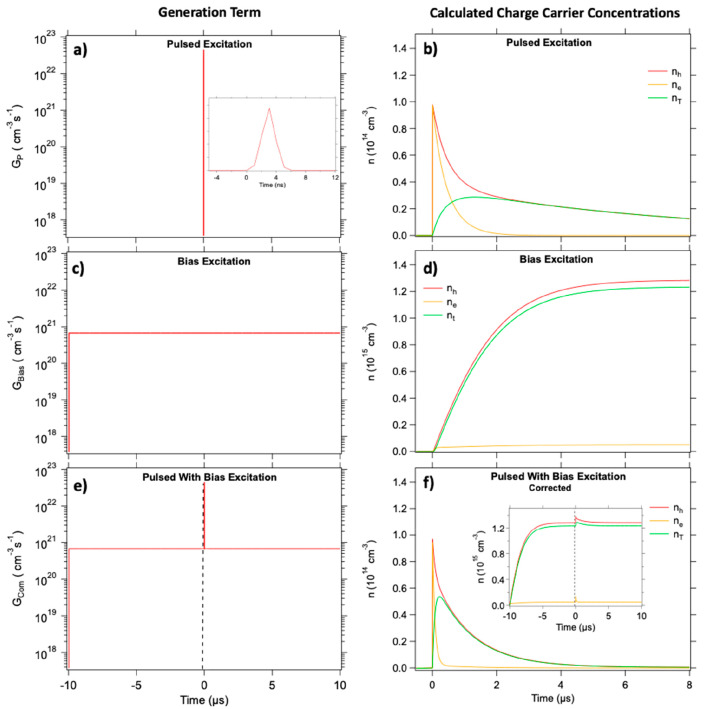
Simulation of the charge carrier concentrations using different illumination sources for a bare MAPbI_3_ layer. When only pulsed excitation is used, the generation term, *G*_P_, represents the temporal generation profile of the laser pulse and its intensity (here 9.8 × 10^13^ charges/cm^3^ per pulse) shown in panel a, which results in the electron (orange), hole (red), and trapped carrier (green) concentrations shown in panel b. The steady-state concentrations can be modeled with a continuous generation profile, as represented in panel c, leading to the modeled concentrations shown in panel d. For the analysis of the TRMC traces in the presence of bias illumination, the two generation terms are combined to *G*_Com_ as shown in panel e. After subtraction of the DC contribution, we can extract the relevant concentrations, as presented in panel f. The magnitude of *G*_Bias_ for the bias illumination is derived from the LED power as described in the Supporting Information.

The above model and fitting procedure were applied to the three systems under investigation, i.e., bare MAPbI_3_, MAPbI_3_/C60, and MAPbI_3_/Spiro-OMeTAD for many laser intensities. The protocol to obtain values for the various parameters by fitting the TRMC traces is described in the Supporting Information. The TRMC results (dashed lines) and corresponding fits (solid lines) are shown in Figure 3, while the kinetic parameters are reported in Table 1. The iterative and global analysis of the three systems in parallel allows a detailed and accurate quantification of the optoelectronic properties of MAPbI_3_. The fact that the excitation profile and corresponding charge diffusion within the MAPbI_3_ have no effect on the TRMC signal justifies the use of homogeneous differential equations on a clearly nonhomogeneous system. Electron and hole mobilities in MAPbI_3_ were found to be 30 and 25 cm^2^/(Vs), respectively, in agreement with previously reported values.^24^ Furthermore, in the absence of BI, the results revealed the presence of 10^14^ cm^–3^ deep trap states, *N*_T_, in bare MAPbI_3_, while the dark carrier concentration, *p*_0_, was found to be 6.5 × 10^14^ cm^–3^ (Figure 3a and Table 1). For laser intensities yielding an excess charge density < *N*_T_, the traces overlap, while for higher intensities, second-order recombination becomes the dominant factor in the decay kinetics.

**Table 1:** Rate Constants and Trap Densities Used for the Fits to the TRMC Signals of Bare MAPbI_3_, MAPbI_3_/C60, and MAPbI_3_/Spiro-OMeTAD without and with Bias Illumination

	without bias			with bias		
	MAPbI_3_	MAPbI_3_/C60	MAPbI_3_/ Spiro	MAPbI_3_	MAPbI_3_/ C60	MAPbI_3_/ Spiro
*k*_2_ (×10^–9^ cm^3^/s)	2.0	2.0	1.8	2.0	2.0	1.8
*k*_T_ (×10^–9^ cm^3^/s)	9.8	9.8	9.8	9.8	9.8	9.8
*k*_D_ (×10^–9^ cm^3^/s)	0.20	0.20	0.20	0.20	0.20	0.20
*N*_T_ (×10^14^ cm^–3^)	1.0	1.0	1.0	22	3.0	22
*p*_0_ (×10^14^ cm^–3^)	6.5	6.5	0.6	6.5	6.5	0.6
*k*_ext_ (×10^6^ cm^3^/s^−1^)		9.0	15		1.0	8.0
*k*_rec_ (×10^6^ cm^3^/s^−1^)		0.20	0.23		0.20	1.0

**Figure 3:**
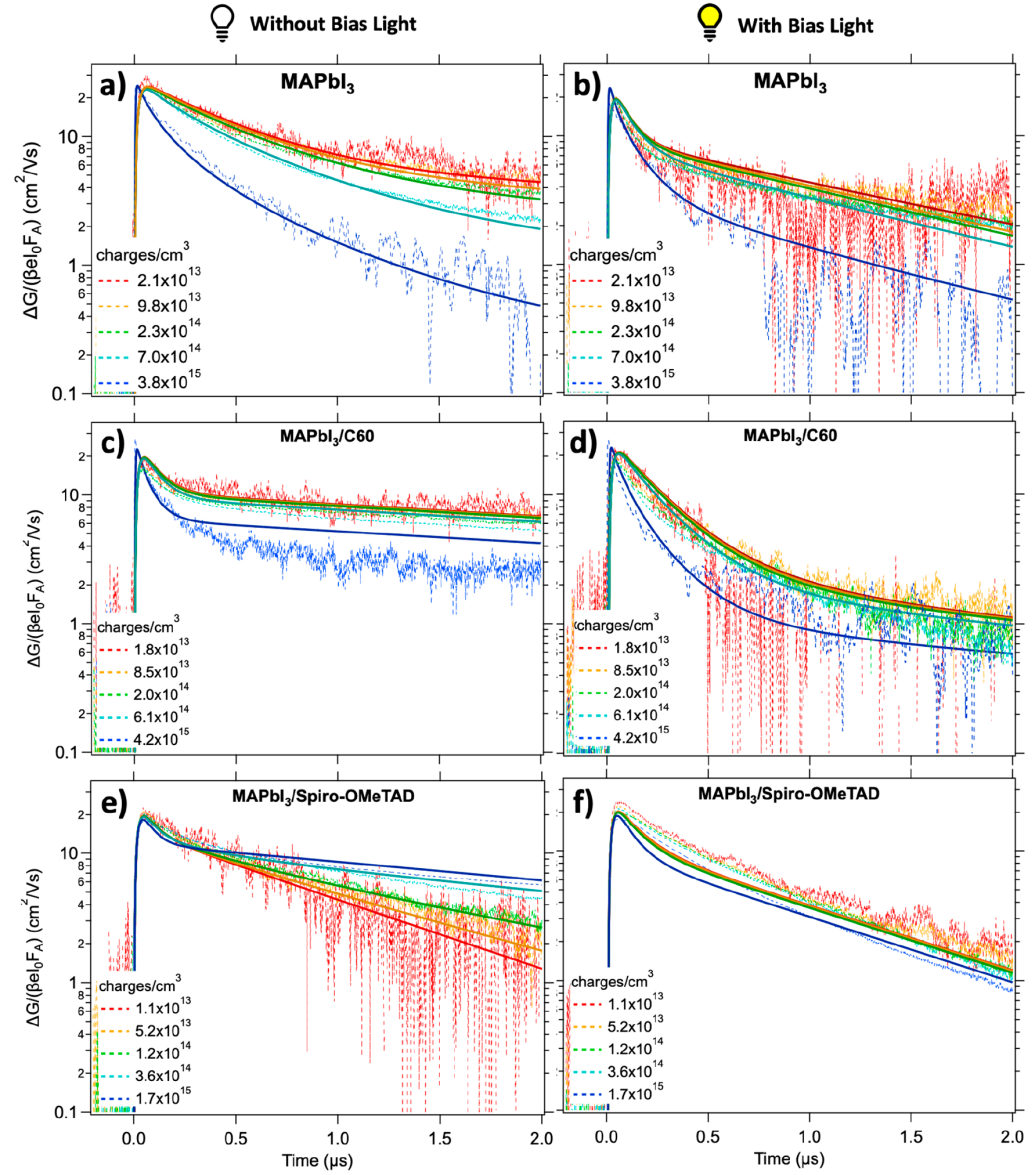
TRMC traces (dashed lines) and fits (solid lines) upon 650 nm pulsed excitation without (left) and with (right) bias illumination for MAPbI_3_ single layer (a and b), MAPbI_3_/C60 (c and d), and MAPbI_3_/Spiro-OMeTAD (e and f).

Interestingly, we observed a strong reduction of *p*_0_ to a value of 6.0 × 10^13^ cm^–3^ in the MAPbI_3_/Spiro-OMeTAD bilayer (Figure 3e), while for MAPbI_3_/C60 no change in *p*_0_ was observed. This can be explained by assuming that the Fermi level of the MAPbI_3_ is below that of Spiro-OMeTAD. Note that the Spiro-OMeTAD in this study was not intentionally doped nor exposed to oxygen. Equilibration of the Fermi levels on contacting MAPbI_3_ and Spiro-OMeTAD leads to transfer of positive carriers to the latter. This implies that our bare MAPbI_3_ layer deposited on quartz is to some extent p-doped, which is in line with recent studies.^29,30^ To corroborate this explanation, we performed steady-state microwave conductance (SSMC) measurements on bare and bilayers in the dark. An SSMC experiment provides information on the background conductivity of a semiconductor layer.^31^ On comparison of the MAPbI_3_ to the MAPbI_3_/Spiro-OMeTAD bilayer, we observed an appreciable reduction of the background conductivity in the bilayer (see the Supporting Information, Figure S4). In analogy to the reasoning above on equilibration of the Fermi levels, holes are transferred from the MAPbI_3_ to the Spiro-OMeTAD layer. Because of the decreased hole mobility in the Spiro-OMeTAD, an overall reduction in conductivity is expected in agreement with our SSMC experiments. This result supports the conclusion regarding the type of doping in MAPbI_3_. Apart from this change in *p*_0_ for the MAPbI_3_/Spiro-OMeTAD bilayer, we can use for fitting the bilayers the kinetic parameters found for the MAPbI_3_ layer completed by introduction of a first-order extraction and a recombination process (see Table 1). Interestingly the presented kinetic model captures the reversed intensity dependencies for both bilayers very well (compare panels c and e of Figure 3). For the MAPbI_3_/Spiro-OMeTAD, a higher intensity leads to longer lifetimes, while for the MAPbI_3_/C60, higher intensities lead to faster decays. As previously discussed,^23,27^ the different behaviors are related to the nature of the deep trap states in MAPbI_3_, which have been proven to be electron traps.

Now we turn to the experiments with BI and start with the bare MAPbI_3_ shown in Figure 1c. The fast recombination is followed by a small but long-lived tail. Fits on the TRMC signals with BI (Figure 3b) reveal that, most importantly, the same parameters can be used except for the trap state concentration, *N*_T_, which increases by more than 1 order of magnitude, reaching 22 × 10^14^ cm^–3^. This increment can be related to ionic motion in the MAPbI_3_ film, induced by the BI. Interestingly after storage of the MAPbI_3_ film in the dark for 3 h, *N*_T_ reverts back to its original value, which demonstrates the reversibility of this degradation process (see Figure S7a in the Supporting Information). Taking this light instability into account, the model can accurately fit the TRMC traces without any further modification of the fitting parameters given in Table 1, as can be seen from Figure 3b.

As mentioned, BI of the MAPbI_3_/C60 bilayer leads to decay kinetics comparable to those of MAPbI_3_ under BI. However, the MAPbI_3_/C60 bilayer does not show any sign of a tail (see Figure 2c) implying no major increase in *N*_T_ under BI; that is, *N*_T_ does not substantially vary in strong contrast with the value found in bare MAPbI_3_. In fact, from the fitting of the TRMC traces of the MAPbI_3_/C60 bilayer with BI, we observe only a minor increment of the trap state concentration, reaching 3 × 10^14^ cm^–3^. Hence, we can conclude that the C60 layer hinders the formation of additional deep trap states under BI, presumably at the surface of the MAPbI_3._ This is in line with previous research arguing that C60 is able to passivate the surface of perovskite materials, resulting in efficient charge carrier extraction and high device efficiencies.^8−10,23^ From Table 1 we notice that the only parameter that changes under BI is *k*_ext_. We suggest that *k*_ext_ is reduced under BI because of pilling up of electrons in the C60. This leads to formation of an electric field over the interface, impeding the extraction process of electrons into C60.

For the MAPbI_3_/Spiro-OMeTAD bilayer measured under BI, a combination of both effects is observed. In contrast to MAPbI_3_/C60 bilayers, the presence of Spiro-OMeTAD does not prevent the degradation process leading to an increase of *N*_T_. Furthermore, the extraction process is reduced by the presence of the bias illumination, in combination with a 4-fold increase of *k*_rec_ (see Table 1). As previously mentioned, the instability of MAPbI_3_ under BI can be related to ionic motion in the perovskite layer. During BI of the MAPbI_3_/Spiro-OMeTAD bilayer, holes are collected and accumulate in the HTL, attracting negatively charged iodine ions toward the interface. Ion accumulation at the interface has been indicated as one of the reasons of hysteresis in the *J*–*V* curves^5,7,9,21^ and could explain the changes in extraction (reduced) and interfacial recombination (increased) rate constants. In addition, TRMC measurements performed after the BI was turned off showed much faster decay kinetics, which only partially recovered after 24 h, as shown in Figure S7c. It has been reported that some of the iodine ions can chemically interact with the Spiro-OMeTAD molecule, leading to irreversible degradation of the HTL,^18^ in line with the only partial recovery that we observe. The results presented here provide a strong link between the surface/interface properties and the instability of the perovskite under steady-state illumination.

Although the model presented in Scheme 1 might not include all processes in full detail, the combined set of processes can be used to describe the main trends of the charge carrier dynamics in MAPbI_3_ and bilayers under BI. Knowing the rate constants enables us to calculate a number of interesting aspects, including the various loss factors and the quasi Fermi level splitting (QFLS) under simulated sun light. We calculated the steady-state carrier concentrations described by the coupled differential equations. The generation term, *G*_Bias_, comprises the intensity corresponding to illumination of the MAPbI_3_ layer with 0.3 suns (see the Supporting Information for the calculation). The evolution of the various concentrations in time until steady-state is achieved is shown in Figures S6. Furthermore, the calculated concentrations are compared to the results of the SSMC experiments for MAPbI_3_ and MAPbI_3_/C60 samples (see Figure 4a for MAPbI_3_ data). From the fits (solid lines) to the data points (markers) the concentration of charge carriers as a function of the light intensity can be derived (see Figure 4b).^24,32^ The SSMC results on MAPbI_3_/Spiro-OMeTAD have not been included because of the light instability of the bilayer at higher illumination densities. In Figured 4b, these SSMC results are compared with the calculated concentrations of mobile charges at 0.3 suns, showing perfect agreement. However, the calculated values at 1 sun are a factor of 2–3 too high, exceeding 2.0 × 10^15^ cm^–3^ in MAPbI_3_ and 3.0 × 10^15^ cm^–3^ in the bilayer (data points not shown). For MAPbI_3_, this can be attributed to the intensity-dependent light degradation, which makes the *N*_T_ value extracted at 0.3 suns inaccurate for simulations at higher intensities. In addition, for MAPbI_3_/C60 it is likely that at higher intensities the extraction and recombination rates become even closer.

**Figure 4:**
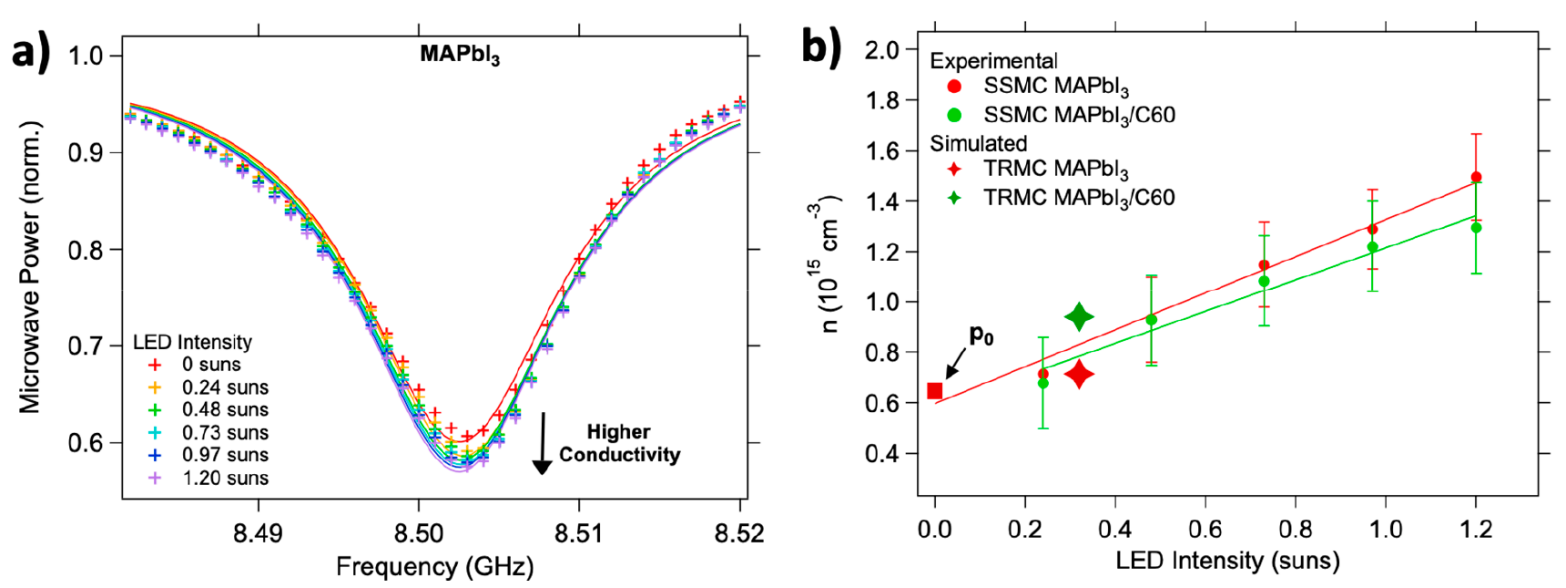
(a) Evolution of the steady-state microwave conductance dip under various LED light intensity values. The experimental data points are indicated by the markers. The solid lines represent the fits from which the conductivity and corresponding carrier concentrations have been derived. A decrease of the normalized microwave power is related to an increased conductivity in the sample, i.e., higher charge carrier concentration under illumination. (b) Charge carrier concentrations in MAPbI_3_ (red) and MAPbI_3_/C60 (green) experimentally determined by SSMC measurements (circles) are compared to the simulated concentrations at 0.3 suns (diamonds), showing excellent agreement. The background concentration of charge carriers, *p*_0_, determined from the fits is also shown as a red square at the LED intensity of 0 suns.

In view of the fact that our model can accurately describe the concentrations at 0.3 suns, the losses for each decay channel can now be quantified and are presented in Table 2. For MAPbI_3_, the decay via trap-mediated recombination is rather large mainly because of the substantial rise in *N*_T_ related to the light instability of MAPbI_3_ and absence of a surface passivating agent. For the MAPbI_3_/C60 bilayer, we notice that the charge extraction is limited (<25%) and the carriers mainly decay in the MAPbI_3_ bulk via second-order and trap-assisted recombination. In contrast, hole extraction and back recombination dominates the kinetics in the MAPbI_3_/Spiro-OMeTAD bilayer. The majority of the light-induced holes (75%) decays by getting first extracted by the Spiro-OMeTAD followed by recombination with excess electrons. Despite the higher concentration of trap states under bias illumination, trap-assisted recombination is relatively small because of the fast saturation of almost all *N*_T_, followed by slow recombination.

**Table 2:** Calculated Loss Fractions Using the Kinetic Parameters from the TRMC Fits at 0.3 suns^a^

	second order (%)	trap-assisted (%)	charge extraction (%)	populated *N*_T_ (%)	QFLS (eV)
MAPbI_3_	29 (97)	71 (3)		56 (93)	1.12 (1.16)
MAPbI_3_/C60	54 (36)	11 (3.5)	23 (60.5)	84 (45)	1.15 (1.13)
MAPbI_3_/Spiro	16 (40)	8 (0.30)	75 (59.7)	99 (100)	1.11 (1.13)

a The values are obtained using the “with bias” fitting parameters of Table 1. The values in parentheses are obtained using the “without bias” parameters and are provided for comparison.

From the concentration curves calculated and shown in Figures S6, also the quasi Fermi level splitting, QFLS, corresponding to the upper limit of *qV*_OC_ of a device, can be calculated using
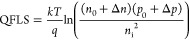
6In eq 6, *k* is the Boltzmann constant, *T* the temperature, and *q* the elementary charge; *n*_0_ and *p*_0_ represent the thermal equilibrium concentrations of electrons and holes, respectively, while Δ*n* and Δ*p* are the photoexcited excess charge carrier concentrations. The intrinsic carrier concentration, *n*_i_, has been evaluated to be ca. 1 × 10^5^ cm^–3^. The calculated QFLS values in bare MAPbI_3_, in MAPbI_3_/C60, and in MAPbI_3_/Spiro-OMeTAD bilayers under 0.3 suns are reported in Table 2. A larger splitting is observed in MAPbI_3_/C60 than for the bare MAPbI_3_, while a clear reduction is obtained for MAPbI_3_/Spiro-OMeTAD. This can be expected on the basis of the C60 passivation effect and ionic motion toward the Spiro-OMeTAD interface mentioned in the above discussion. For the latter, reducing the value of *k*_rec_ is a logical step in order to improve the *V*_oc_. Therefore, designing TLs giving rise to an active heterojunction capable of accepting one type and repelling the counter charge is essential. Alternatively, reduction of *k*_rec_ could be achieved, for example, by introducing an interlayer between the perovskite and TL.^33−36^ Furthermore, careful tuning of the band offsets might be a way to influence the rate constants involved.

Lastly, our study reveals that the determination of the kinetic parameters under bias illumination is of utmost importance if we want to understand the loss mechanisms under device operation. Since some of the kinetic parameters are varying with light intensity in MAPbI_3_ and MAPbI_3_/TL, these parameters cannot be used for the simulation of device performance directly. As given in parentheses in Table 2, the loss fractions and QFLS values are substantially different in case the “without bias” parameters of Table 1 are used for the steady-state simulation at 0.3 suns. In MAPbI_3_, the strong underestimation of the trap density would lead to an underestimation of the trap-assisted recombination contribution, ultimately leading to a higher *V*_OC_. For the bilayers, the interfacial processes are the most affected by BI. Despite the passivation effect of C60, the electron extraction in MAPbI_3_/C60 is substantially reduced under BI. For the MAPbI_3_/Spiro-OMeTAD, the ionic migration toward the interface with Spiro-OMeTAD enhances the interfacial recombination under BI. Moreover, as concluded from the SSMC results, these effects are likely to be even more pronounced at higher illumination intensities.

To summarize, in this Letter we performed time-resolved microwave photoconductance measurements in the presence and absence of bias illumination in single and bilayers. A kinetic model was used to describe the kinetics, including the charge generation and charge recombination and extraction. Owing to the iterative analysis of the three systems using a broad range of laser intensities, we were able to accurately determine the kinetic parameters for bare MAPbI_3_, MAPbI_3_/C60, and MAPbI_3_/Spiro-OMeTAD in the presence and absence of BI. For MAPbI_3_ we found that the same parameters can be used under BI except for the trap state concentration, *N*_T_, which increases by more than 1 order of magnitude, reaching 22 × 10^14^ cm^–3^. This increment is related to ionic motion in the MAPbI_3_ film. Interestingly, after storage of the MAPbI_3_ film in the dark for 3 h, *N*_T_ reverts back to its original value, which demonstrates the reversibility of this degradation process. For the MAPbI_3_/C60 bilayer under BI, we observe only a minor increment of *N*_T_, which shows that C60 hinders the formation of additional deep trap states. Under BI, only the extraction rate *k*_ext_ is reduced because of pilling up of electrons in the C60, which leads to formation of an electric field over the interface, impeding the extraction process of electrons into C60.

In contrast to MAPbI_3_/C60 bilayers, the presence of Spiro-OMeTAD does not prevent the degradation process leading to an increase of *N*_T_. Furthermore, under BI the extraction process is reduced, in combination with a 4-fold increase of *k*_rec_, which are related to ion accumulation at the interface. Moreover, we show that to calculate carrier concentrations for deducing device parameters, like the QFLS, it is important to use the kinetic parameters found under BI, which is in particular relevant for the MAPbI_3_/Spiro-OMeTAD bilayer. This study adds to the understanding of both the heterojunctions’ interfacial properties as well as the origin of the light instability in MAPbI_3_, crucial factors for the performance of perovskite-based devices.

## Experimental Methods

The TRMC is a contactless technique, whose working principle is based on the interaction of microwaves with photoexcited charge carriers.^37^ In a TRMC measurement, the sample is illumined by a short laser pulse (3 ns fwhm, 10 Hz) and the light-induced excess charge carriers absorb a small part of the microwave power resulting in a slight reduction of the reflected microwave power, which is monitored by a sensitive microwave detection system. The DC part of the signal is electronically subtracted using an offset regulator, and the remaining AC signal is amplified by a broadband amplifier (GHz–kHz). The changes in measured microwave power, Δ*P*(*t*), are related to the changes in photoconductance, Δ*G*(*t*), by
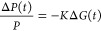
7where *K* is a predetermined sensitivity factor.^37^ The recombination pathways revealed by TRMC are not limited to the radiative recombination observed by commonly applied photoluminescence spectroscopy (PL) nor by higher-order recombination processes as in transient absorption (TA). Furthermore, with a slow repetition rate of 10 Hz, all the charges in the sample have relaxed back to the original state before the next pulse arrives. A similar TRMC analysis was conducted to extract the charge carrier dynamics under continuous illumination using a white LED. With a semitransparent mirror the sample was simultaneously illuminated with the pulsed laser and the LED. The intensity of the latter was 6.6 mW (ca. 0.3 suns in the visible region), leading to the generation rate of approximately 6.7 × 10^20^ charges/cm^3^ per second. Reproducibility within a single batch of MAPbI_3_ layers is very good, and variations in signal height and lifetimes are typically less than 20%; between batches prepared using the same equipment, the variations increase but are within 50%.

## Supporting Information Available

Experimental details regarding sample preparation, bias illumination, the microwave conductance technique, and the modeling and fitting protocols (PDF)
