# Supplementary material for: Quantifying Charge Carrier Recombination Losses in MAPbI3/C60 and MAPbI3/Spiro-OMeTAD with and without Bias Illumination
Source: J Phys Chem Lett. 2022 Aug 10;13(32):7523–31. doi: 10.1021/acs.jpclett.2c01728 (PMC9393883; doi:10.1021/acs.jpclett.2c01728)
Supplement: Supplementary file 1 — jz2c01728_si_001.pdf [file jz2c01728_si_001.pdf]

Supporting Information

Quantifying Charge Carrier Recombination Losses in $MAPbI_3$/C60 and $MAPbI_3$/Spiro-OMeTAD with and without Bias Illumination

V.M. Caselli, and T.J. Savenije*

Department of Chemical Engineering, Delft University of Technology, van der Maasweg 9, 2629 HZ Delft, the Netherlands

E-mail: T.J.Savenije@tudelft.nl

Sample Preparation

215 nm thick $MAPbI_3$ thin films have been prepared by spin-coating a 37 wt% solution of MAI (synthetized following standard procedure[1]) and $Pb(Ac)_2{\cdot}3H_2O$ (Sigma-Aldrich) powders (3:1 ratio) in DMF. The solution was spin-coated on plasma cleaned quartz plates at 2000 rpm for 45 s in a nitrogen filled glovebox and left to dry for 15 minutes at room temperature. The films have been annealed at 100°C for 5 minutes.

Selective transport materials have been deposited on top of the $MAPbI_3$ films via the following procedures: 30 nm C60 layer has been thermally evaporated, while a 75 mg/mL solution of Spiro-OMeTAD in chlorobenzene has been spin-coated at 1500 rpm for 45 s. The samples been then dried for 1 hour at 60°C to remove the remaining solvent.

The XRD spectrum of the $MAPbI_3$ thin film is shown in **Figure S1**. XRD measurements have been performed with a *Brüker D8* diffractometer (Co Kalfa-1, 1.78Å)

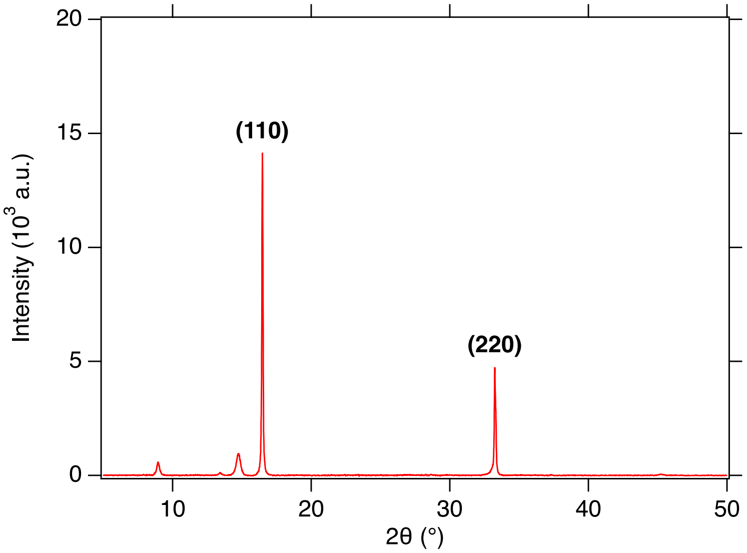

Figure S1: $MAPbI_3$ thin film XRD spectrum.

Bias Illumination

To determine the generation profile, G_{Bias}, for fitting the TRMC measurements recorded with bias illumination, the following procedure was used as also described by Guo et al.[2] First the light intensity of the white light LED (see emission spectrum in **Figure S2a**) at the sample position has been measured with a Silicon photodiode (*Coherent, OP-2/LM-2 VIS*) yielding a value of 13.5 mWatt cm^{-2}. This intensity matches to the total number of integrated photons of the white light LED. Next, we integrated the number of photons over the wavelength emission range of the LED, however now corrected for the fraction of absorbed photons yielding values for G_{Bias} of 6.74, 6.79 and 6.46×10^{20} cm^{-3}s^{-1} in the pristine $MAPbI_3$, $MAPbI_3$/C60 and $MAPbI_3$/Spiro-OMeTAD, respectively. The absorbance spectra are shown in **Figure S2b**.
To relate the observed values of G_{Bias} to AM1.5 we corrected the LED spectrum for the solar emission spectrum and the absorbance spectrum of the photoactive layer (shown in Figure **S2c**). After integration this corrected emission spectrum over the wavelength, the G_{Bias} profile corresponding to AM1.5 is found.

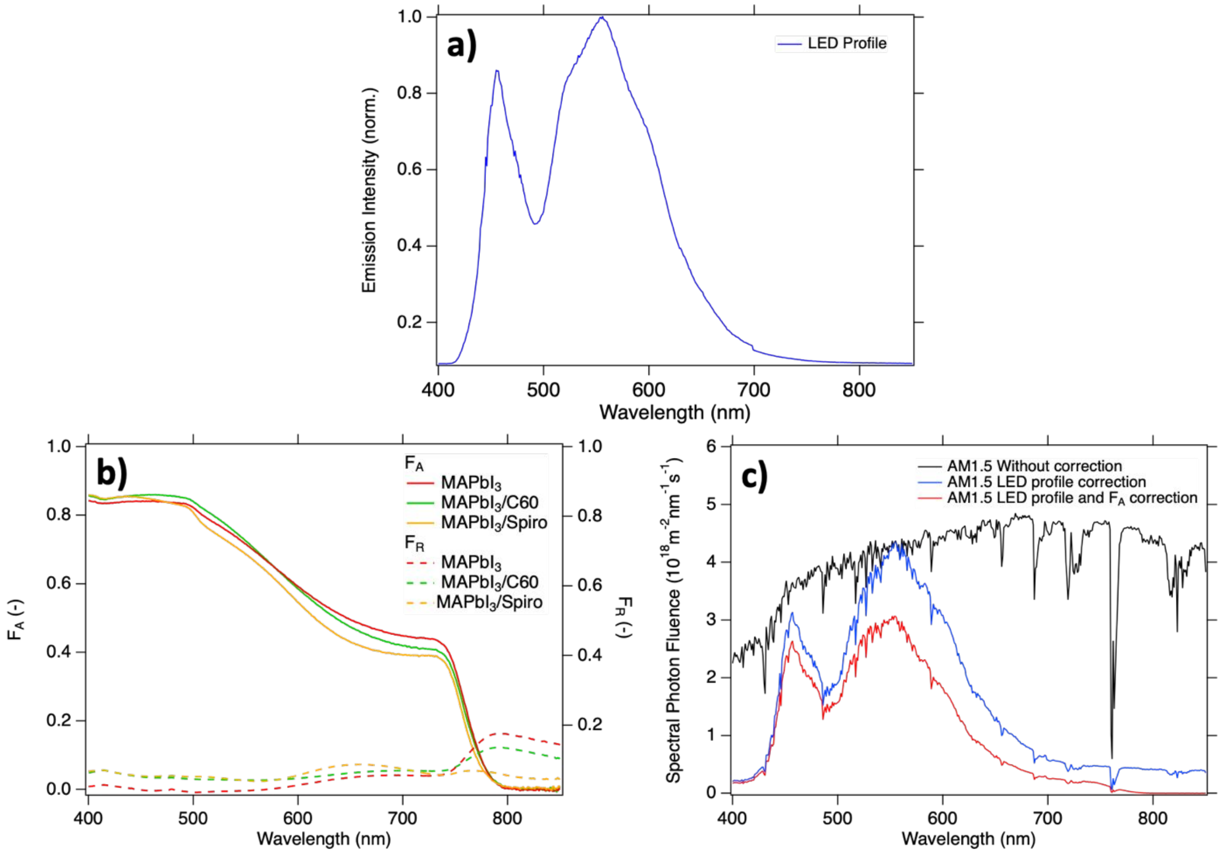

Figure S2: a) LED emission profile; b) fraction of absorbed (solid lines), F_A, and reflected (dashed lines), F_R, of pristine $MAPbI_3$ (red), $MAPbI_3$/C60 (green), and $MAPbI_3$/Spiro-OMeTAD (orange) films; c) AM1.5 spectra not corrected (black), corrected for LED emission profile (blue), and F_A of the $MAPbI_3$ sample used in this study.

Microwave Conductance Technique

A schematic representation of the microwave conductance set-up is shown in **Figure S3**. While an accurate description of its working principles can be found elsewhere,[3] it is of relevance to underlying fundamental distinctions during the different operation modes applied in this study. Steady-state microwave conductance (SSMC) measurements have been conducted in the dark and in presence of bias illumination only. In the former case, it is possible to extract information regarding the background conductivity of the system under investigation. This is done by performing a scan of the microwave power over a broad frequency range (8.2-12.2 GHz) in a resonant cavity, and analyzing the dip of microwave power at the resonance frequency. In fact, the deep observable at the resonance frequency is not only related to the cavity's properties, but also to the dimensions, dielectric properties and conductivity of the sample analyzed. In a previous study we have demonstrated how it is possible to extract the conductivity, σ, by fitting the resonance dip.[4] Furthermore, steady-state concentration of photogenerated charge carriers can be calculated from the conductivity measurements during steady-state illumination, as it was done in the study presented by Guo et al.[5] In this paper, we have performed SSMC measurements in the dark, see **Figure S4**, and in presence of bias illumination, as shown in **Figure 4d in the main text**. From **Figure S4** it is possible to notice a higher microwave power detected at the resonance frequency for $MAPbI_3$/Spiro-OMeTAD compared to pristine $MAPbI_3$. As discussed in the main text, this is an indication of reduced background conductivity in the bilayer. On the other hand, from the fittings of the SSMC results under bias illumination we have estimated the conductivity of the $MAPbI_3$ layer, and estimated the number of charge carriers, *n*, in the material according to:

$$n = \frac{\sigma}{e\Sigma\mu} \qquad \text{(Eq. S1)}$$

where e is the elementary charge and Σμ the sum of electron and hole mobilities. From this analysis it is not possible to directly discriminate the electron and hole contributions. Nonetheless, we can use the estimated values to validate the TRMC model, as described in the main text.

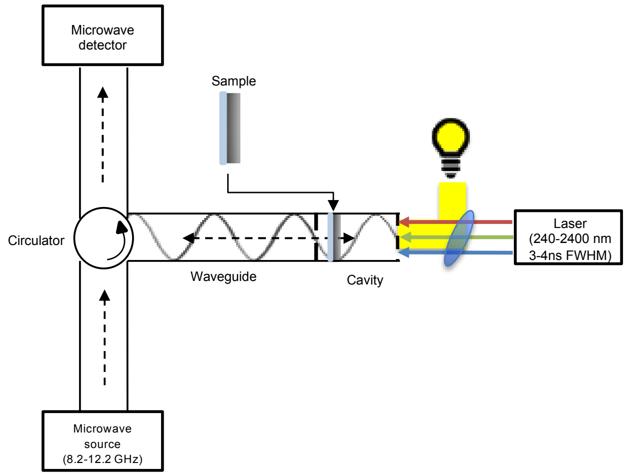

Figure S3: Schematic representation of the TRMC set-up for bias light experiments. During SSMC measurements, either no light is shone on the sample, or the LED light is directly in front of the cell. During pulsed experiments only, the LED is turned off, while both illuminations are simultaneously reaching the sample during the bias experiment with the help of a semi-transparent mirror.

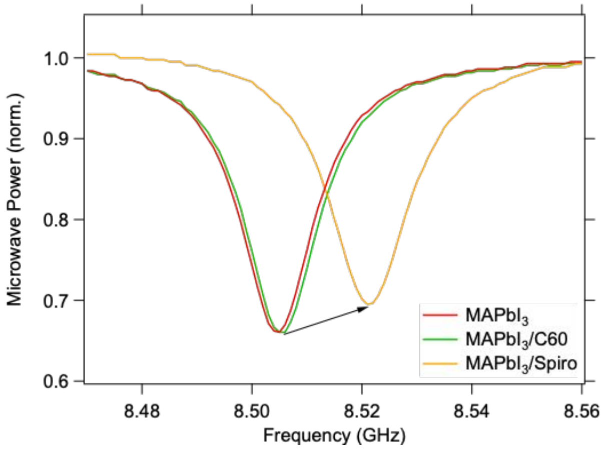

Figure S4: SSMC results in the dark for pristine MAPbI3 (red), $MAPbI_3$/C60 (green), and $MAPbI_3$/Spiro-OMeTAD (orange).

The homogeneity of excitation throughout the sample has been tested by performing the same experiments upon FS illumination, i.e. from the perovskite and TM side. The results are shown in **Figure S5**. The lower magnitude of $MAPbI_3$/C60 sample in **Figure 5b** compared to the single layer can be explained by the increased fraction of absorbed bias light in the TM layer, which is now directly irradiated.

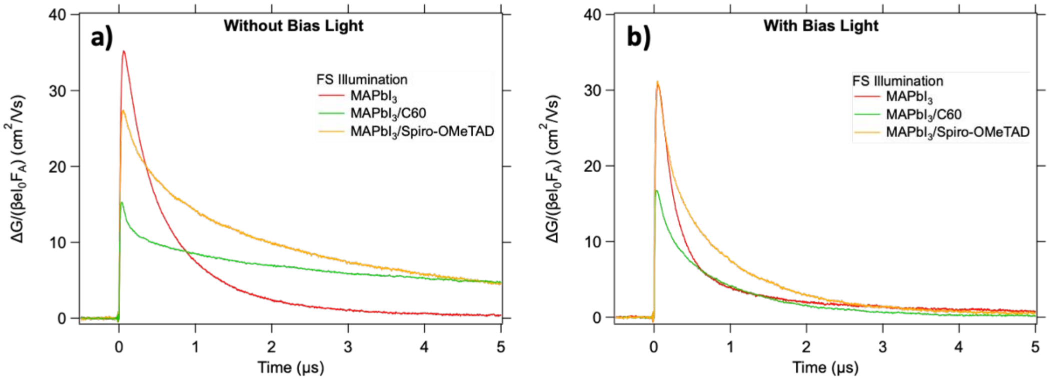

Figure S5: $MAPbI_3$ (red), $MAPbI_3$/C60 (green) and $MAPbI_3$/Spiro-OMeTAD traces recorded at 650 nm FS illumination (ca. $7x10^9$ photons/cm2) a) without and b) with bias illumination.

Modelling TRMC measurements

Similar to equations 2-5 in the main text, the differential equations used to fit the TRMC traces of the $MAPbI_3$/Spiro-OMeTAD heterojunction are:

$$\frac{dn_e}{dt} = G_P - k_2 n_e (n_h + p_0) - k_T n_e (N_T - n_T) - k_{rec} n_e \quad \text{(Eq. S2)}$$

$$\frac{dn_h}{dt} = G_P - k_2 n_e (n_h + p_0) - k_D n_t (n_h + p_0) - k_{ext} n_h \quad \text{(Eq. S3)}$$

$$\frac{dn_T}{dt} = k_T n_e (N_T - n_T) - k_D n_T (n_h + p_0) \quad \text{(Eq. S4)}$$

$$\frac{dn_{HTL}}{dt} = k_{ext} n_h - k_{rec} n_e \quad \text{(Eq. S5)}$$

To come to the photoconductance the excess electrons and holes are multiplied by their mobilities according to

$$\Delta G = e\beta L (n_e \mu_e + n_h \mu_h) \quad \text{(Eq. S6)}$$

Global, iterative fitting procedure

In absence of bias illumination (only pulsed excitation, G_P):

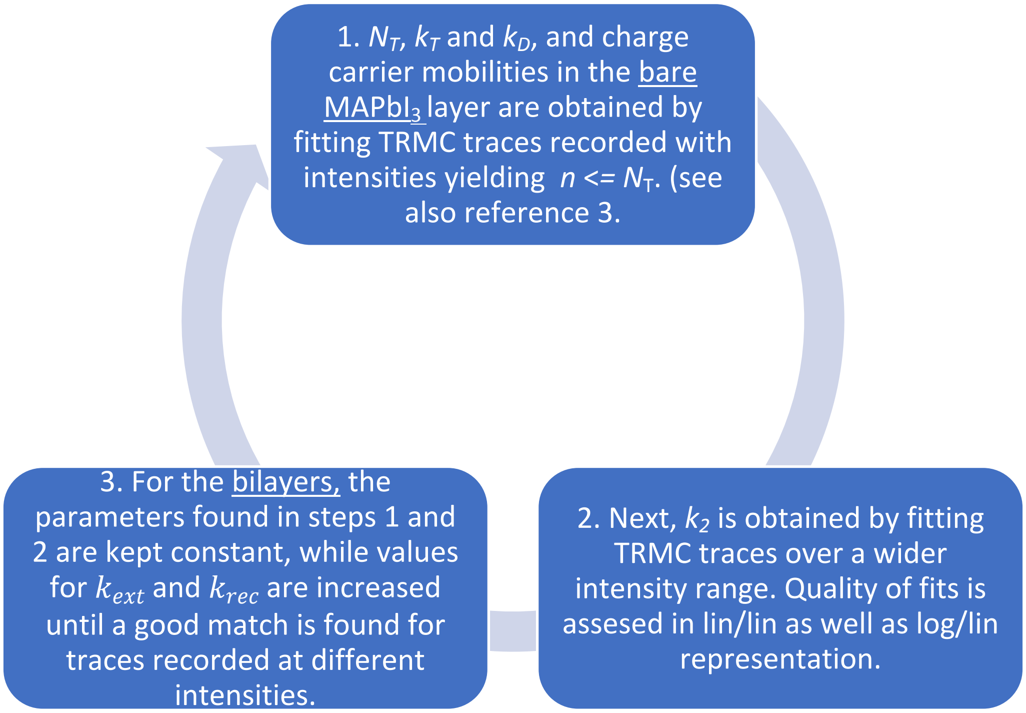

In presence of bias illumination (G_{com}):

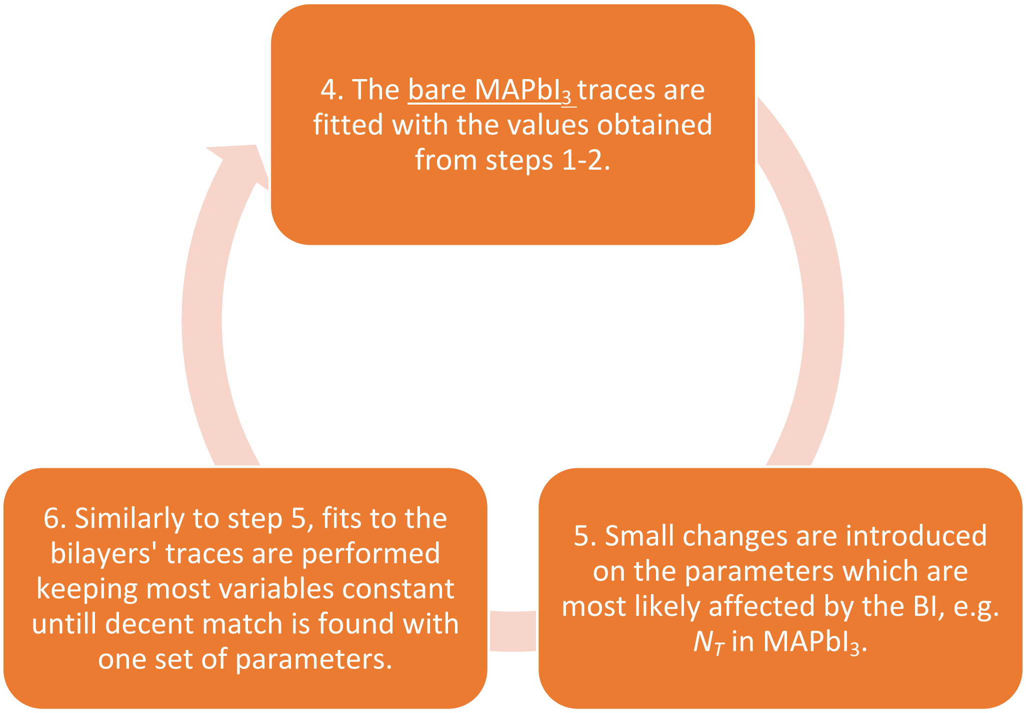

Time dependent charge carrier concentrations

The time-dependent concentrations obtained from a steady state simulation at 0.3 suns are shown in **Figure S6** for $MAPbI_3$ (**a**), $MAPbI_3$/C60 (**b**) and $MAPbI_3$/Spiro-OMeTAD (**c**).

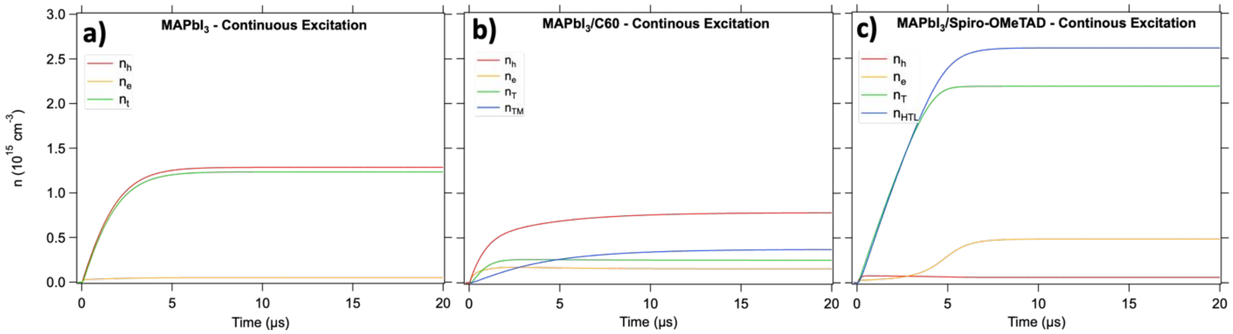

Figure S6: Simulated time-dependent concentrations under continuous (0.3 suns) illumination of free electrons and holes, n_e and n_h, trapped carriers, n_T and transferred electrons/holes into the selective transport materials, $n_{ETL/HTL}$, in $MAPbI_3$ (a), $MAPbI_3$/C60 (b), and $MAPbI_3$/Spiro-OMeTAD (c).

Lastly, the bias illumination effects during and after illumination are shown **Figure S7** for $MAPbI_3$ (a), $MAPbI_3$/C60 (b), and $MAPbI_3$/Spiro-OmeTAD (c).

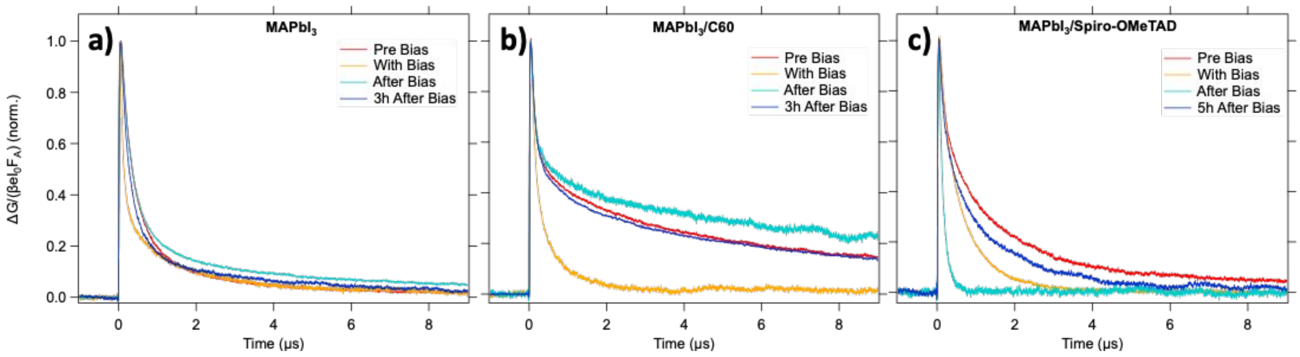

Figure S7: Normalised TRMC traces recorded at a laser intensity of ca. $3.5x10^{14}$ cm^{-3} per pulse, showing the effect of bias light on a) $MAPbI_3$, b) $MAPbI_3$/C60 and c) $MAPbI_3$/Spiro-OMeTAD bilayer during and after illumination. No further changes have been observed in the next 24h.

References

(1) Kim, H. S.; Lee, C. R.; Im, J. H.; Lee, K. B.; Moehl, T.; Marchioro, A.; Moon, S. J.; Humphry-Baker, R.; Yum, J. H.; Moser, J. E.; et al. Lead Iodide Perovskite Sensitized All-Solid-State Submicron Thin Film Mesoscopic Solar Cell with Efficiency Exceeding 9%. *Sci. Rep.* **2012**, *2*, 1–7.

(2) Guo, D.; Caselli, V.; Hutter, E. M.; Savenije, T. J. Comparing the Calculated Fermi Level Splitting with the Open Circuit Voltage in Various Perovskite Cells. *ACS Energy Lett.* **2019**, 0–5.

(3) Savenije, T. J.; Guo, D.; Caselli, V. M.; Hutter, E. M. Quantifying Charge-Carrier Mobilities and Recombination Rates in Metal Halide Perovskites from Time-Resolved Microwave Photoconductivity Measurements. *Adv. Energy Mater.* **2020**, *10*, 1–12.

(4) Caselli, V. M.; Fischer, M.; Meggiolaro, D.; Mosconi, E.; De Angelis, F.; Stranks, S. D.; Baumann, A.; Dyakonov, V.; Hutter, E. M.; Savenije, T. J. Charge Carriers Are Not Affected by the Relatively Slow-Rotating Methylammonium Cations in Lead Halide Perovskite Thin Films. *J. Phys. Chem. Lett.* **2019**, *10*, 5128–5134.

(5) Guo, D.; Caselli, V. M.; Hutter, E. M.; Savenije, T. J. Comparing the Calculated Fermi Level Splitting with the Open-Circuit Voltage in Various Perovskite Cells. *ACS Energy Lett.* **2019**, *4*, 855–860.
